# Electrophysiological correlates of distance and direction processing during cognitive map retrieval: A source analysis

**DOI:** 10.3389/fnhum.2023.1062064

**Published:** 2023-02-22

**Authors:** Mélanie Teixeira De Almeida, Martin Seeber, Katherina Gschwend, Roland Maurer, Igor Faulmann, Nicolas Burra

**Affiliations:** ^1^Faculty of Psychology and Educational Science, University of Geneva, Geneva, Switzerland; ^2^Functional Brain Mapping Laboratory, Department of Fundamental Neurosciences, Campus Biotech, University of Geneva, Geneva, Switzerland

**Keywords:** spatial navigation, memory, cognitive map, retrieval, source analysis, EEG

## Abstract

**Introduction:**

The cognitive map is an internal representation of the environment and allows us to navigate through familiar environments. It preserves the distances and directions between landmarks which help us orient ourselves in our surroundings. The aim of our task was to understand the role played by theta waves in the cognitive map and especially how the cognitive map is recalled and how the manipulation of distances and directions occurs within the cognitive map.

**Method:**

In order to investigate the neural correlates of the cognitive map, we used the Cognitive Map Recall Test, in which 33 participants had to estimate distances and directions between familiar landmarks tailored to their own knowledge. We examined the role of theta waves in the cognitive map, as well as the brain regions that generated them. To that aim, we performed electroencephalographic source imaging while focusing on frequency spectral analysis.

**Results:**

We observed increases of theta amplitude in the frontal, temporal, parahippocampal gyri and temporal poles during the recall of the cognitive map. We also found increases of theta amplitude in the temporal pole and retrosplenial cortex during manipulation of directions. Overall, direction processing induces higher theta amplitude than distance processing, especially in the temporal lobe, and higher theta amplitude during recall compared to manipulation, except in the retrosplenial cortex where this pattern was reversed.

**Discussion:**

We reveal the role of theta waves as a marker of directional processing in the retrosplenial cortex and the temporal poles during the manipulation of spatial information. Increases in theta waves in frontal, parahippocampal, temporal and temporal pole regions appear to be markers of working memory and cognitive map recall. Therefore, our Cognitive Map Recall Test could be useful for testing directional difficulties in patients. Our work also shows that there are two distinct parts to the cognitive map test: recall and manipulation of spatial information. This is often considered as two similar processes in the literature, but our work demonstrates that these processes could be different, with theta waves from different brain regions contributing to either recall or manipulation; this should be considered in future studies.

## Introduction

Spatial navigation consists of the ability to find our way in our surroundings (Golledge, 1999), but how we use this ability daily to orient ourselves has been of interest over the years. Tolman (1948) proposed the concept of having a cognitive map as an internal representation of the environment preserving the spatial relationships between places. This means that places that are closer to the environment will also be closer to the cognitive map (Epstein et al., 2017). In order for the cognitive map to be useful for spatial navigation, it must contain representations of distances and the directions of the different places relative to one another (Epstein et al., 2017; Lisman et al., 2017). According to the cognitive map theory, it would allow us to plan a route from one location to another, using shortcuts and planning novel routes (Epstein et al., 2017). To be able to access the cognitive map, the individuals must have had an extensive exploration of their environment or have studied a cartographic map (Zhang et al., 2014). When exploring their environment, one person can view it from two different perspectives, also called spatial reference frames (SRF): egocentric and allocentric. An allocentric spatial reference frame characterizes the relationship that objects have among themselves, independently of the subject's location in regard to these objects. The egocentric spatial reference frame characterizes the relationship between the subject, having itself as a reference, and the objects of its environment (Jeffery, 2003). Exploration of the environment provides individuals with predominant egocentric representations at first, when using locomotion to move in the environment (Jeffery, 2003). Indeed, individuals explore the environment by collecting spatial relationships between landmarks, which implies that it has not yet built an allocentric knowledge of it, hence limiting their representation of space to a predominantly egocentric SRF. Following the model of Siegel and White (1975), individuals will first gain a representation of landmarks, and after some exploration, they will progressively have a representation of the routes between landmarks. These landmarks will act as an anchor to connect between map coordinates (Epstein et al., 2017). Finally, they will learn the configurations of landmarks and routes with the distances and directions relative to one another, meaning an allocentric representation, composing the cognitive map, which will allow planning a route to a goal destination (Siegel and White, 1975; Epstein et al., 2017).

Interestingly, when investigating the mechanisms of the cognitive map, studies have often considered recalling and manipulation of the cognitive map as a similar process, rather than a two-way process (Rosenbaum et al., 2004; Spiers and Maguire, 2007b; Descloux and Maurer, 2018). The following studies have attempted to shed light on the neural correlates of the cognitive map, often using functional magnetic resonance imaging (fMRI). However, even though fMRI provides useful and detailed anatomical information about brain areas involved in the cognitive map processes, its low temporal resolution does not allow the observation of cognitive processes that take place more rapidly than the hemodynamic response. For example, the hippocampus has been implicated in playing a role in the cognitive map, especially when navigating through complex and detailed environments (Maguire et al., 2006), but not for simpler and schematic representations of environments learned long ago (Burgess et al., 2002). The parahippocampus is additionally involved in the processing of landmarks (Janzen and Van Turennout, 2004), which is essential for large-scale navigation as it helps not only in orienting to the surroundings but also in forming and using the cognitive map. Moreover, the parahippocampus encodes allocentric distances and directions of landmarks (Burgess et al., 2001). This same brain region, along with the entorhinal cortex and the medial temporal lobe (MTL), is involved with tracking Euclidean distance (meaning, the distance between two points) (Howard et al., 2014).

The parietal cortex, apart from MTL, plays an important role in navigation, regarding the generation and maintenance of egocentric representations of an environment (Byrne et al., 2007). Other studies have pinpointed the parietal cortex to be involved in remembering and imagination of familiar scenes from an egocentric point of view (Spiers and Maguire, 2007b). Therefore, the parietal cortex seems to be of major importance during the building and recalling process of the cognitive map, especially during the first-person exploration of the environment and when remembering it from an egocentric perspective. The parietal cortex also seems to encode directions, as some studies have highlighted its possible role in enabling the body to orient in the correct direction, along a path, in an egocentric reference frame (Byrne et al., 2007; Epstein et al., 2017). Spiers and Maguire (2007a) additionally reported a significant correlation between parietal cortex activity with egocentric directions to goals in a virtual environment.

The prefrontal cortex is involved in different cognitive functions such as planning, decision-making, and working memory (Purves et al., 2004; Funahashi, 2017). Studies have proposed that the role of the anterior prefrontal cortex would be to manipulate information while keeping goal information in mind (Koechlin et al., 1999; Grieves and Jeffery, 2017). It should be noted that the dorsolateral and anterolateral prefrontal regions have been shown to be involved in other forms of memory, such as the retrieval of long-term declarative memories (Purves et al., 2004). Hence, the prefrontal cortex probably participates in remembering locations of the cognitive map and its manipulation, along with the other brain regions mentioned above.

The retrosplenial cortex is another key structure supporting navigation; it was suggested by Vann et al. (2009) to be implicated in scene translation from egocentric to allocentric reference frames and vice versa (Epstein et al., 2017). Moreover, the retrosplenial cortex activates during mental navigation and scene viewing of familiar places (Epstein, 2008). On the other hand, the retrosplenial cortex is involved in direction processing, as shown by patients with retrosplenial damage, they are able to recognize places and landmarks but unable to use it for directional purposes, even in familiar environments (Epstein, 2008).

Despite these insights provided by fMRI studies about the cognitive map, it lacks crucial information regarding fast-changing cognitive processes that fMRI cannot measure. However, electroencephalography (EEG) is more indicated to that aim, since it allows us to discover the fast-changing temporal dynamics of brain information processing circuitry, due to its excellent temporal resolution (millisecond level). To that aim, frequency analysis is indicated, as it allows observing the changes in brain waves under a specific time window corresponding to the process that we expect to observe.

One particular frequency range, theta (4–8 Hz), has gained attention over the years because of its involvement in spatial navigation tasks. Theta waves have been linked to several cognitive processes of particular relevance for this study, such as the retrieval of object location, egocentric navigation, and allocentric direction processing (White et al., 2012; Kaplan et al., 2014; Lin et al., 2015). However, less is known about the involvement of theta waves when an individual operates on their own cognitive map. Spatial cognition is highly dependent on self-motion cues, hence studying it in a static setting (as is necessary for MRI or EEG) is especially challenging, and even more so when considered from an ecological perspective. In this study, our aim was to understand the role played by theta waves in the cognitive map, especially how the cognitive map is recalled and how manipulation of distances and directions within the cognitive map occurs. For that purpose, we use the same ecological paradigm as Descloux and Maurer (2018), where participants had to recall and judge between distances and directions of well-known landmarks based on their own cognitive maps, therefore theoretically manipulating their cognitive map.

We examine whether there is a difference between the process of recall and manipulation of the cognitive map of familiar environments. As there are limited studies comparing these two processes, we present studies that best approach our hypothesis. A study by Oberauer (2002) demonstrated that active recalled information held in working memory (i.e., manipulation) takes more time to process than passive recall. For this reason, we can expect the manipulation of the cognitive map to have greater theta activity during our task.

To further investigate the neural correlates of distance and direction processing, we aim to identify the brain regions that contribute to these mechanisms in the theta band. A study by Bischof and Boulanger (2003) showed an increase in cortical theta during directional changes and also with maze difficulty. We could, therefore, imagine direction having greater theta as it might be more complex to process than distance.

Regarding the recalling of the cognitive map during our task, we expect task-related theta increases in parietal, parahippocampal, frontal, and temporal regions, according to the following and previous studies. As stated earlier, the implication of the parietal cortex in recalling scenes from an egocentric point of view (Spiers and Maguire, 2007b), as well as the implication of the parahippocampus in landmark processing and scene construction of imagined scenes in fMRI studies (Rosenbaum et al., 2004; Hassabis et al., 2007; Chrastil, 2013), leads us to suppose a theta increase in those regions. Kaplan et al. (2014) also showed the involvement of increased theta amplitude and synchrony between the medial prefrontal cortex and the right anterior medial temporal cortex during the retrieval of objects' locations. Moreover, in a working memory task, Jensen and Tesche (2002) demonstrated that frontal theta activity increased, according to an increasing working memory load. Therefore, according to previous studies, we propose that parietal, frontal, temporal, and parahippocampal regions would increase theta when recalling the cognitive map, especially in direction processing involving additional landmarks, hence increasing the number of items to remember.

As for the manipulation of the cognitive map, we expect frontal, retrosplenial, and parietal theta increases during direction processing. An interesting study by Lin et al. (2015) in a virtual navigation task showed that the parietal and retrosplenial cortex theta activity correlated with performances in directional navigation. They also found that the retrosplenial cortex activity in the theta band was strongly correlated with direction pointing in allocentric navigators. Glahn et al. (2002) showed that manipulation of spatial information activated more the dorsolateral–prefrontal cortex than simple maintenance, even when increasing memory loads. Consequently, we expect those brain regions to have higher theta activity when manipulating directional information. However, we expect more activation in the theta band for the temporal cortex and parahippocampus during the manipulation of distances because these areas are related to computing Euclidean and allocentric distances, respectively (Burgess et al., 2001; Epstein et al., 2017).

## Materials and methods

### Participants

For this study, 33 participants (*N* = 15 women and *N* = 18 men) were recruited, of whom 29 were right-handed and four left-handed. No power analysis was performed. However, we aimed to recruit a number of participants similar to the study by Faulmann et al. (2020), i.e., 23 participants. Participants were young and healthy (*Mean* = 23.13, *SD* = 3.18, from 18 to 31 years old) with no neurological disorders and came from all socioeconomic backgrounds. In order to control a high familiarity with the city, all had lived in the city of Geneva for more than 2 years. We assumed that people familiar with it have built a strong, stable cognitive map. Participants who were volunteers did not receive any monetary compensation, while participants who were students earned course credits with their participation. This study was approved by the Ethics Committee of the University of Geneva, and informed consent was obtained for all participants.

### Stimuli and apparatus

In our task, participants had to estimate distances and directions between pairs of well-known landmarks, using the Cognitive Map Recall Test (CMRT) developed by Descloux and Maurer (2018). For the task, we presented 40 trials comprising 20 questions of distance and 20 questions of direction, as described below. Our training session comprised 12 trials (six questions of distance and six questions of direction).

Distance questions required one starting point (i.e., base location) landmark and two other landmarks. The base location changed across trials and across participants, as it was tailored to each participant's personal knowledge of the city. In this condition, participants were asked to imagine being at the base location and to determine which of the two other landmarks was the furthest from that base location. Questions were generated by means of a custom program, CMRT-Gen (Maurer, 2016), with the constraints that the distance between the pairs of landmarks to evaluate were in a ratio of 1.4 and 1.6. This ratio corresponded to a clear, though not obvious, difference in distance between the two landmarks.

Direction questions required one starting point, a second location defining a direction from the starting point, and two other landmarks. In this condition, participants had to imagine themselves at the base location, looking toward the second location, and to judge, between a pair of landmarks, for which one they had to turn more to look toward it. The same CMRT-Gen program was used to generate the questions, with the constraints that, in order to be faced, one landmark would require a rotation larger by 45°-60° than the other landmarks. Moreover, there was a minimum of 45° between the least excentric landmark and the base direction. In this condition, pairs of landmarks could be presented on the same side (monodirectional) or on each side of the base location (bidirectional). We tried to balance monodirectional and bidirectional questions by having approximately the same number of each type.

### Procedure

Two weeks before the EEG recording, all participants were asked to provide a written list of 25 well-known landmarks of the city of Geneva. The landmarks had to be precise and must have been visited on foot by the participants. We instructed participants to choose landmarks that played an important role in how they oriented themselves in the city. For each landmark given by the participants, the exact geographic coordinates were extracted using the Swisstopo website (https://map.geo.admin.ch/) and the coordinate tool therein, which yields coordinates, in meters, in the Swiss MN03 system. Based on the landmarks provided by the participants and their coordinates, two types of questions were built: distance and direction.

Participants first filled out a consent form for this experiment and were advised that they could stop the experiment at any time. They also completed a socio-demographic questionnaire before being placed in a Faraday's cage for the EEG recording. Prior to EEG recordings, they were instructed to avoid any movements.

Participants performed a training session for 5 min and then the task itself for 20 min. The training session did not have any passing criteria because the aim was for participants to only familiarize themselves with the task. The task started right after the training session and ensuring participants did not have questions and understood the task. For both training and task, questions were presented on screen using E-prime (Psychology Software Tools, Pittsburgh, PA) with lights off and at ~80 cm distance from the computer. For training and task, questions were randomized and alternated between distance and direction questions. At the beginning of each question and at the beginning of each answer, a trigger was sent systematically to record participants' answers and time spent on the question and answer screen. They received the same instructions for the training and for the task, but the brain activity was recorded only during the task.

### Electrophysiological recording and analysis

We used a BioSemi Active Two system (http://www.biosemi.com; BioSemi B.V., Amsterdam, Netherlands) configured to the 10–20 electrode system. In this active electrode system, the quality of electrode contact with the skin was evaluated by the offset relative to the magnitude of the feedback loop formed by the CMS-DRL electrodes, which was held below 30 mV throughout the recording. The electrodes were connected to an AD-Box ActiveTwo amplifier with a sampling rate of 1,024 Hz. For the Active Two system, the online filter is low-pass only and performed by the A DC's decimation filter with a fifth-order sync response with a −3 dB point at 1/5th of the selected sample rate (refer to http://www.biosemi.com/faq/adjust_filter.htm). Sixty-four electrodes were placed on the scalp and six additional electrodes on the face: two placed 1 cm at the outer canthi, one above and under the right eye, and two on each ear lobes.

For the pre-processing, we used Brain Vision Analyser 2.2 (Brain Products, Gilching, Germany). The signal was then off-line re-referenced to both earlobes. We used Butterworth filters 1 Hz order 2. We also removed the signal corresponding to wrong answers, keeping only the signal from correct answers for analysis. We included a mean of 15.13 (SD = 1.9) and 13.1 (SD = 3.1) trials per condition. We performed an Independent Component Analysis (ICA) to remove eye blinks and saccades influence on the signal.

In the following analyses, we used MATLAB 2018a (The MathWorks, Natick, 2018), and with a customized script, we have transformed the scalp EEG signal into the frequency domain signals using Morlet wavelet transform (2 Hz steps, mother wavelet: 1 Hz center frequency, three cycles full-width half maximum). Electrical source imaging was applied based on a linear forward model based on realistic head models, i.e., a Locally Spherical Model with Anatomical Constraints (LSMAC) (Brunet et al., 2011; Birot et al., 2014), while using a template anatomy of the MNI brain (Collins et al., 1994). The linear distributed inverse solution LAURA (de Peralta Menendez et al., 2004) was used to compute three-dimensional (3D) current density distributions for each solution point of about 5,000 points equally covering the gray matter volume.

In the final steps of the analysis, we used CARTOOL to visualize our data and to compute paired *t*-tests for our analysis. This procedure allowed us to visualize the significative activation at the scalp surface. In order to examine if there was a correspondence between the scalp theta amplitude and the sources that generated them, we then proceeded to analyze the inverse domain signal. We repeated the same procedure as the scalp frequency signal analysis, but this time using Regions Of Interest (ROI). According to our hypotheses, our ROIs included the temporal gyri, the parahippocampal gyri, the parietal gyri, the retrosplenial cortex, and the dorsolateral prefrontal gyri, based on the MNI atlas. We then extracted the theta source amplitude of each ROIs for each participant, condition, and interval. Intervals were divided into question-locked and response-locked. Question-locked is the part where participants were asked about distance or direction questions, with a minimum of 5,000 ms interval. Response-locked is the part where participants had to answer the previous question, again with a minimum of 5,000 ms interval.

## Results

Single-subject FFT and source analysis are available on the Open Science Framework (OSF) at https://osf.io/37bde/. The following analyses were performed with Jamovi (version 1.0) at a *p*-value < 0.05 and the graphs were made with Rstudio. Three participants were discarded due to poor general performance at the task (accuracy < 0.60). Analyses were then performed on 30 participants.

### Behavioral results

**Figure 1:**
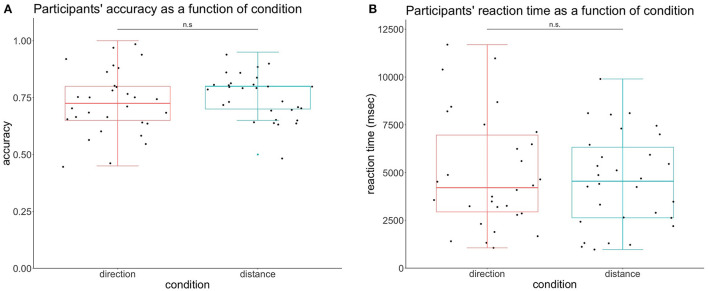
Participants' behavioral performance according to the condition. **(A)** Participants' accuracy per condition. The accuracy in the condition direction was not significantly different from the condition distance. **(B)** Participants' reaction time per condition. The reaction time in the direction condition did not reach the different levels of significance.

We wanted to test if what we observed in electrophysiological results could be explained by behavioral results. To this aim, we performed a paired *t*-test to test for differences in the accuracy of response participants depending on the condition, and it was not significant, *t*_(29)_ = −0.924, *p* = 0.36. The condition direction had the accuracy of Mean = 0.73, SD = 0.14 and the condition distance had the accuracy of Mean = 0.76, SD = 0.097, represented in Figure 1A.

We also looked for differences in the reaction time (RT) depending on the condition with a dependent *t*-test. This analysis revealed no significant differences between the two modalities *t*_(29)_ = 0.849, *p* = 0.40. RT was calculated for corrected answers only and all units are in milliseconds. The reaction time of distance was Mean = 4601, SD = 2467, and the RT of direction was Mean = 4988, SD = 2974, represented in Figure 1B.

### EEG results

#### Theta frequency domain results

**Figure 2:**
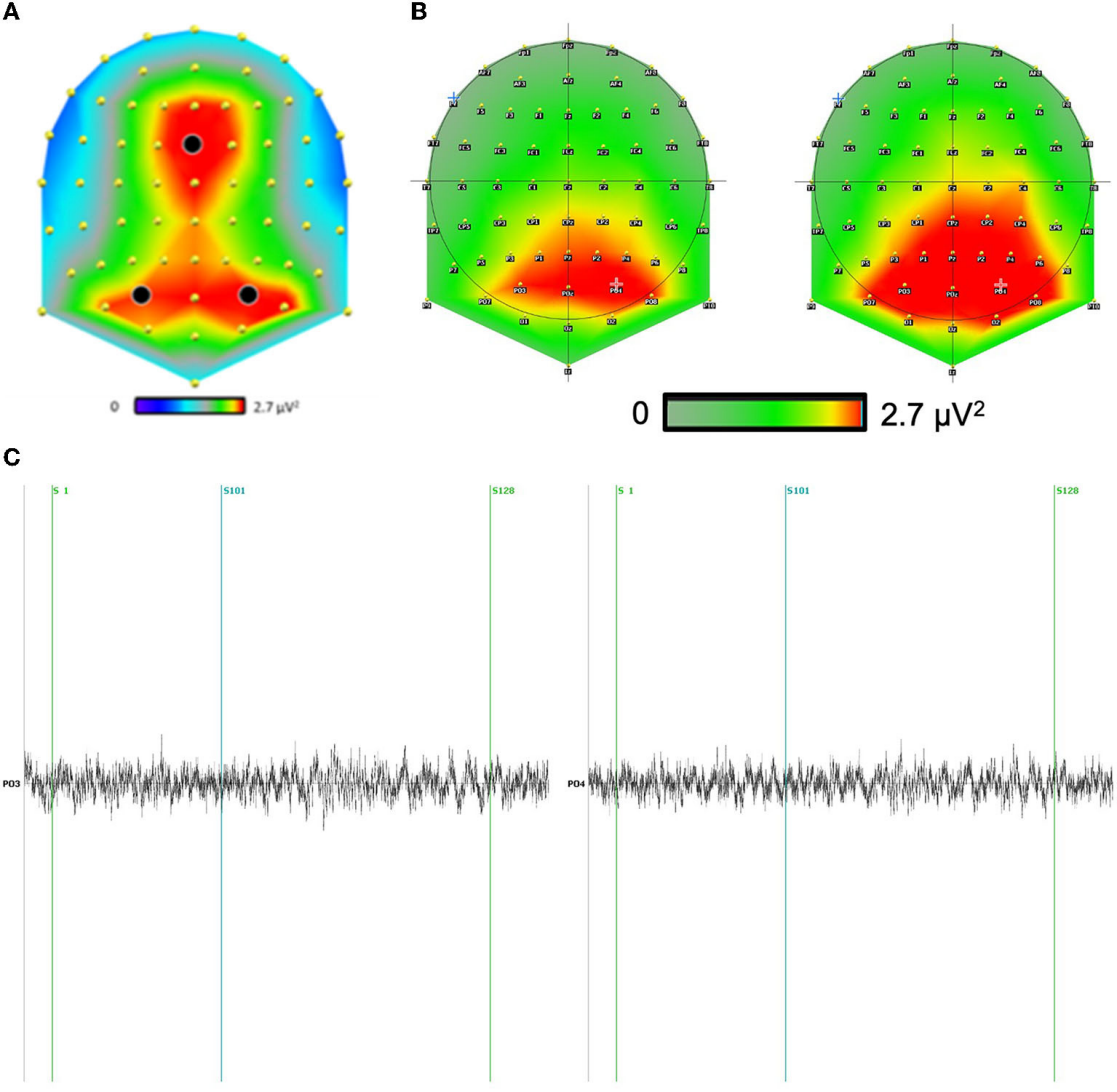
**(A)** The theta scalp distribution revealed two strong clusters of activity: one in the frontal site and one in the bilateral parieto-occipital sites. **(B)** We included the scalp theta distribution for both conditions above. We have focused our analysis on the electrodes with the largest differences. Indeed, the main difference between conditions is mainly a global increase of the theta signal in the direction condition as compared to distance condition, independently of sites. **(C)** Example of a typical raw channel focused on PO3-PO4 (maximum effect) during question distance (S1) and response distance (S101) for a correct answer (S128).

For the scalp analysis, we performed two analyses: one on the frontal site (FCz) and one on the bilateral parieto-occipital sites (in black in Figure 2), where the theta activity was maximal irrespective of conditions.

A 2 × 2 × 3 repeated-measures ANOVA, with the factors condition (distance and direction), interval of the experiment (question-locked or response-locked), and electrodes (Fcz, P03, P04), revealed a main effect of condition, *F*_(1,29)_ = 146.79, *p* < 0.001, where the theta activity was larger for the direction condition (Mean = 2.9 μV^2^, SD = 0. 65) than the distance condition (Mean = 2.67 μV^2^, SD = 0. 64). We also measured an interaction effect condition × interval of the experiment, *F*_(1,29)_ = 4.69, *p* = 0.034.

#### Theta source domain results

For this analysis, we performed a 2 × 2 × 2 × 5 repeated-measures ANOVA, with the factors condition (distance and direction), interval of the experiment (question-locked or response-locked), hemisphere (left or right), and brain regions (parietal, frontal, temporal, temporal pole, parahippocampal gyri). We tested the effects of the different factors on the theta mean activity (TMA). All values are in μA/mm^3^·Hz.

#### The main effect of the condition

We had a significant main effect of the condition on the TMA, *F*_(1,29)_ = 23.741, *p* < 0.001. The condition direction was greater than the condition distance. The distance condition was Mean = 1.81 × 10^−3^, SD = 6.29 × 10^−4^, while the direction condition was Mean = 1.99 × 10^−3^, SD = 7.19 × 10^−4^.

#### The main effect of interval

We also had a significant main effect of the interval on the TMA, *F*_(1,29)_ = 9.104, *p* = 0.005. The question-locked was greater than the response-locked. For the question-locked, we had Mean = 1.93 × 10^−3^, SD = 7.12 × 10^−4^ and for the response-locked we had Mean = 1.87 × 10^−3^, SD = 6.49 × 10^−4^.

#### The main effect of brain regions

There was a significant main effect of brain regions with Greenhouse–Geisser correction applied, *F*_(2.17, 62.95)_ = 35.562, *p* < 0.001. We could observe the parietal, parahippocampal, and frontal regions being smaller than the temporal pole and the temporal region. The parietal region was Mean = 1.58 × 10^−3^, SD = 4.59 × 10^−4^, the temporal region was Mean = 1.93 × 10^−3^, SD = 6.35 × 10^−4^, the temporal pole was Mean = 2.53 × 10^−3^, SD = 8.94 × 10^−4^, the parahippocampus was Mean = 1.68 × 10^−3^, SD = 3.46 × 10^−4^ and the frontal region was Mean = 1.80 × 10^−3^, SD = 4.82 × 10^−4^.

#### The main effect of hemisphere

We failed to find a significant main effect of the hemisphere on TMA, *F*_(1,29)_ = 3.157, *p* = 0.086. The right hemisphere was Mean = 1.95 × 10^−3^, SD = 7.30 × 10^−4^, while the left hemisphere was Mean = 1.85 × 10^−3^, SD = 6.26 × 10^−4^.

#### Interaction effect of condition × interval

For interaction effects, we found a significant effect of condition depending on the interval of the experiment, *F*_(1,29)_ = 5.795, *p* = 0.023. We performed a contrast with a Bonferroni correction to test if the differences between the conditions were significant inside each interval. We had a significant effect for the condition distance vs. direction in the question-locked, *t*_(38.3)_ = −5.42, *p* < 0.001. We had Mean = 1.82 × 10^−3^, SD = 6.43 × 10^−4^ for the condition distance and Mean = 2.04 × 10^−3^, SD = 7.59 × 10^−4^ for the condition direction during question-locked. We had a significant effect for the condition distance vs. direction in the response-locked, *t*_(38.3)_ = −3.61, *p* = 0.005. We had Mean = 1.80 × 10^−3^, SD = 6.15 × 10^−4^ for the condition distance and Mean = 1.95 × 10^−3^, SD = 6.74 × 10^−4^ for the condition direction during response-locked. Overall, the question-locked was greater than the response-locked and the direction condition was higher than the distance condition.

#### Interaction effect of brain regions × interval × condition

**Figure 3:**
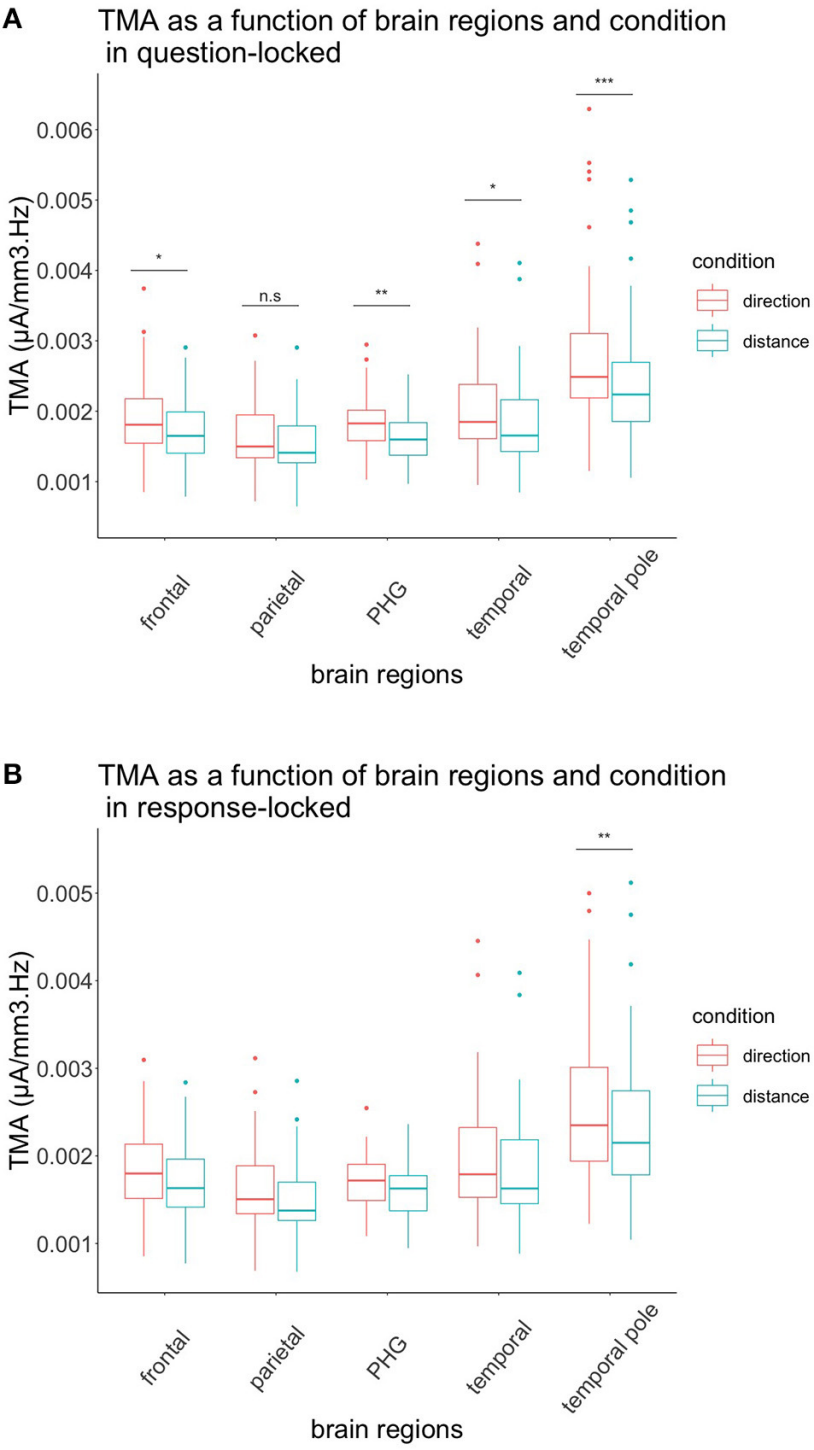
TMA of brain regions and conditions according to their interval. **(A)** Representation of the TMA of each brain region according to their condition in the question-locked. All brain regions, except the parietal region, showed significant differences between distance and direction, with the direction condition being greater than the distance condition. **(B)** Representation of the TMA of each brain region according to their condition in the response-locked. Only the temporal pole showed significant differences between the two conditions, with the direction condition being higher than the distance condition. n.s: *p* > 0.05, *p < 0.05, **p < 0.01, ***p < 0.001.

As depicted in Figure 3, we found a tendential triple effect for the brain regions, depending on the interval and condition of TMA *F*_(1.33, 38.45)_ = 3.677, *p* = 0.051. We also performed contrasts with a Bonferroni correction to test whether the temporal, temporal pole, parietal, parahippocampal, and frontal activation were bigger in the condition direction according to the interval. For the question-locked, we had significant differences between the two conditions for the frontal region with *t*_(77.2)_ = −4.03, *p* = 0.025, for the parahippocampus with *t*_(77.2)_ = −4.44, *p* = 0.006, for the temporal region *t*_(77.2)_ = −4.17, *p* = 0.015, and for the temporal pole with *t*_(77.2)_ = −7.22, *p* < 0.001, while the parietal region was not significant, *t*_(77.2)_ = −2.67, *p* = 1.00. For the distance condition, we had Mean = 1.72 × 10^−3^, *SD* = 4.46 × 10^−4^ for the frontal region; Mean = 1.85 × 10^−3^, SD = 6.10 × 10^−4^ for the temporal; Mean = 2.42 × 10^−3^, SD = 8.50 × 10^−4^ for the temporal pole; Mean = 1.61 × 10^−3^, SD = 3.24 × 10^−4^ for the parahippocampus; and Mean = 1.52 × 10^−3^, SD = 4.29 × 10^−4^ for the parietal region. For the direction condition, we had Mean = 1.92 × 10^−3^, SD = 5.53 × 10^−4^ for the frontal region; Mean = 2.05 × 10^−3^, SD = 6.49 × 10^−4^ for the temporal; Mean = 2.77 × 10^−3^, SD = 1.02 × 10^−3^ for the temporal pole; Mean = 1.82 × 10^−3^, SD = 3.93 × 10^−4^ for the parahippocampus; and Mean = 1.65 × 10^−3^, SD = 4.85 × 10^−4^ for the parietal region. Overall, we observed regions that were greater in the direction condition than the distance condition during the question-locked.

To test our hypotheses in the response-locked experiment, we performed contrasts with a Bonferroni correction. None of them were significant when comparing each region in both conditions, except for the temporal pole, *t*_(77.2)_ = −4.43, *p* = 0.006. For the distance condition, we had Mean = 2.36 × 10^−3^, SD = 7.87 × 10^−4^ for the temporal pole. For the direction condition, we had Mean = 2.57 × 10^−3^, SD = 8.67 × 10^−4^ for the temporal pole. Again, during this interval, the condition direction was greater than the condition distance.

#### ROI analysis: Retrosplenial cortex main effects and interactions

**Figure 4:**
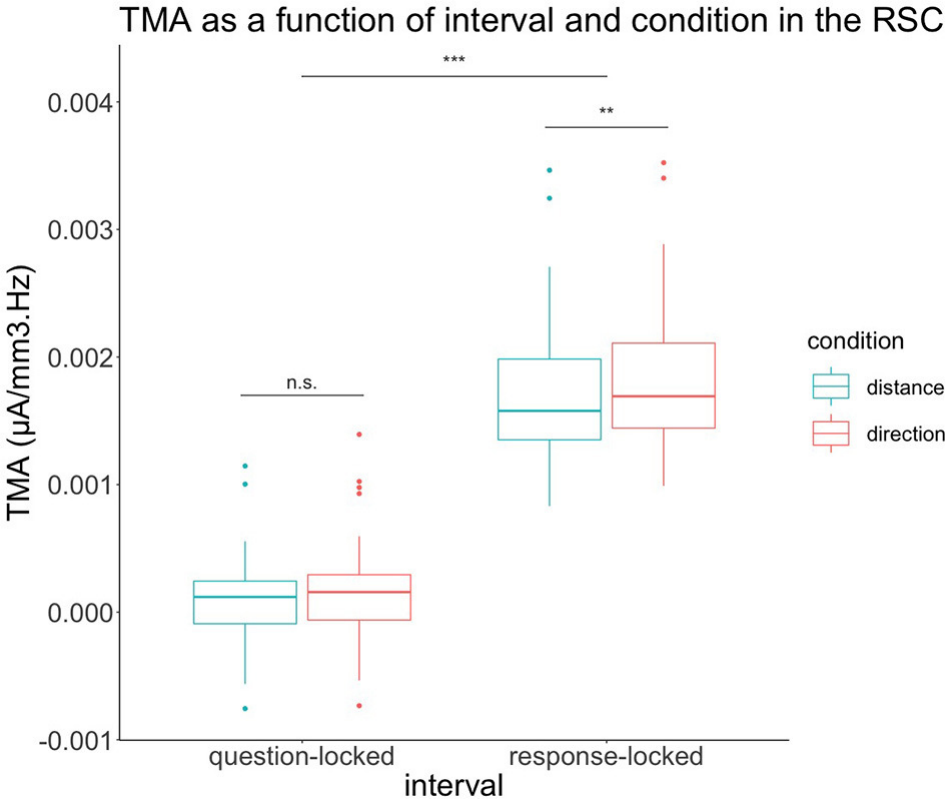
TMA of retrosplenial cortex depending on condition and interval of the experiment. In the question-locked, there were no significant differences in TMA between the two conditions. In response-locked, the TMA of the condition direction is significantly greater than the distance condition. n.s: *p* > 0.05, **p < 0.01, ***p < 0.001.

In order to test our hypotheses regarding the retrosplenial cortex, we performed a 2 (condition) × 2 (interval) × 2 (Brodmann areas 29 and 30) repeated-measures ANOVA in this specific source region, as depicted in Figure 4.

We also had a main effect on the condition for the retrosplenial cortex with *F*_(1,29)_ = 8.24, *p* = 0.008. The direction condition was greater than the condition distance. For the condition, we had Mean = 9.06 × 10^−4^, SD = 9.20 × 10^−4^ for distance and Mean = 1.01 × 10^−3^, SD = 9.83 × 10^−4^ for direction.

Regarding the retrosplenial cortex, we also had a main effect on the interval with *F*_(1,29)_ = 687.67, *p* < 0.001. The response-locked was greater than the question-locked. For the interval, we had Mean = 1.22 × 10^−4^, SD = 3.43 × 10^−4^ for the question-locked and Mean = 1.79 × 10^−3^, SD = 5.45 × 10^−4^ for a response-locked.

The retrosplenial cortex also had an interaction effect between the interval and the condition with *F*_(1,29)_ = 12.07, *p* = 0.002. The retrosplenial cortex was not significant when performing a contrast with Bonferroni correction *t*_(37.1)_ = −1.46, *p* = 0.92 when comparing both conditions during the question-locked, with Mean = 9.44 × 10^−5^, SD = 3.19 × 10^−4^ for the distance condition and Mean = 1.49 × 10^−4^, SD = 3.65 × 10^−4^ for the direction condition. In contrast, the retrosplenial cortex was significant when performing a contrast with Bonferroni correction *t*_(37.1)_ = −3.91, *p* = 0.002 when comparing both conditions during the response-locked with Mean = 1.72 × 10^−3^, SD = 5.16 × 10^−4^ for distance and Mean = 1.86 × 10^−3^, SD = 5.56 × 10^−4^ for direction during the response-locked. During the response-locked, the direction condition was higher than the distance condition.

## Discussion

The aim of this study was to investigate the electrophysiological correlates of spatial navigation, in particular when a person uses a cognitive map. Previous studies have successfully highlighted which brain regions underlie cognitive map processes but failed to account for fast-changing dynamics occurring in the brain during recall and manipulation of a cognitive map. In our study, we addressed this issue using the validated CMRT, in which participants had to remember and manipulate their hometown cognitive map, while EEG recordings were performed to study the link between theta activity (4–8 Hz) and the brain regions supporting cognitive map processes.

### Differences in distance and direction processing

The condition direction demonstrated a higher TMA, but since there were no significant differences in the behavioral performance of participants, we can assume that electrophysiological results were not influenced by task difficulty. This could reflect participants' imagining directional changes, similar to the results by Bischof and Boulanger (2003). This could also be due to a difference in cognitive load, as theta increases with higher working memory demands (Jensen and Tesche, 2002) and complexity (Kahana et al., 1999). Indeed, for the direction condition, the participants had to consider one more landmark: the direction they were facing. This additional landmark can also act as an additional load in the working memory and recall process, resulting in a higher TMA.

### Differences between question-locked and response-locked

Contrary to our prediction and to Oberauer's (2002) study, TMA was higher in the question-locked than in the response-locked. The question-locked interval showing a higher TMA than the response-locked interval could demonstrate a difference in spatial processing between the two intervals of the experiment: cognitive map recall vs. cognitive map manipulation. The recall corresponded to the question-locked in our study, while manipulation occurred in the response-locked of the task. For the question-locked, the cognitive map has to be first recalled and maintained in working memory, and once these elements are integrated, the manipulation occurs in the response-locked. We suppose that the recall process occurring during the question-locked might be more complex than the manipulation, as TMA tends to increase with recall and complexity (Kahana et al., 1999).

### Brain regions involved in the question-locked

For the question-locked, we had the hypothesis of the parietal, frontal, temporal, temporal pole, and parahippocampus to have a higher increase in TMA for the condition direction. This was partially confirmed by our analysis. Indeed, we observed differences between distance and direction processing, with the condition direction showing a higher TMA in all regions. However, there were significant differences between the two conditions in all regions mentioned above, except in the parietal region.

The frontal cortex has been shown to be involved in working memory processes (Harms et al., 2013), and since our task requires the participants to keep in mind the starting location as well as the additional facing landmark for the direction condition, it is not surprising that this region exhibits a higher TMA in the question-locked and direction condition. As mentioned earlier, theta waves tend to augment with increasing memory loads and during recall (Jensen and Tesche, 2002). Frontal regions are also activated during mental imagery in fMRI, which could also explain the high TMA coming from frontal gyri in our task (Ganis et al., 2004).

The parahippocampus showed significant TMA differences for the question-locked between the two conditions, as expected. The involvement of this region in the question-locked might reflect the recall process, since studies have demonstrated the parahippocampus to be implicated in mental imagery, recalling of a familiar area, and scene construction of imagined scenes in fMRI studies (Rosenbaum et al., 2004; Hassabis et al., 2007; Chrastil, 2013). The parahippocampus showing a higher TMA for the direction condition in the question-locked could reflect, as we expected, the retrieval process of landmarks, which is in line with a previous study in fMRI of the CMRT (Faulmann et al., 2020). Indeed, TMA was higher in the direction condition, probably because of the additional landmark.

Surprisingly, the temporal pole has shown the highest TMA, and this region was also the one showing the highest TMA among all. For the question-locked, this might reflect the episodic and retrieval processes of imagining themselves at a specific place. Studies have reported the temporal pole to be involved in episodic memory and visual mental imagery (Luzzatti et al., 1998; Steinvorth et al., 2006), which could explain why TMA was higher in this region. Another interesting point to consider is the fact that TMA in the temporal pole was also higher during direction processing, probably due to the presence of an additional landmark to retrieve (Kaplan et al., 2014).

The temporal region showed significant TMA differences between the conditions in the question-locked. We can explain this as the temporal region being part of the ventral stream of the visual pathway proposed by Milner and Goodale (2008). Since the ventral stream has been shown to be involved in object recognition, it might participate in our task in the recognition of landmarks and their imagination. Furthermore, the fact that TMA was also higher when judging directions could indicate the retrieval of object representations from remote long-term memories (Steinvorth et al., 2006). Again, when judging directions, TMA could be higher due to the presence of one more landmark, increasing the cognitive load (Jensen and Tesche, 2002).

It was, however, quite unexpected that the parietal TMA activity did not change, neither between the two intervals nor between the two conditions. Maybe the participants used the egocentric representations in a similar way for both intervals and conditions, which led to no significant differences between them (Spiers and Maguire, 2007b). It might also be that the parietal gyri were more active in other frequencies during the task that was not investigated.

### Brain regions involved in the response-locked

Parahippocampal differences were not significant between the two conditions for the response-locked, and the means were higher for direction than for distance. A possibility for this observation would be that the parahippocampus codes for allocentric representations of both distance and direction, as demonstrated in a study by Burgess et al. (2001). The parahippocampus could, therefore, be implicated in both direction and distance processing in our task, which did not reflect a significant preference for one over the other in theta frequency.

The frontal gyri failed to show significant differences between the two conditions for the response-locked. We previously mentioned that complexity could play a role in the increase of theta waves; therefore, this might explain the absence of differences in TMA between the two conditions (Kahana et al., 1999). Since there were two landmarks for both conditions, this interval could be of equal complexity. Another possibility would be that the recall process was more demanding on cognitive resources than the manipulation for the response-locked as mentioned above.

Unexpectedly, the temporal pole showed again increased TMA for the response-locked in the direction condition. Lesion studies in the temporal pole have shown impairments in spatial mental imagery and when processing spatial allocentric long-term memories (Luzzatti et al., 1998; Feigenbaum and Morris, 2004). Both of these processes are probably involved in cognitive map processing, but judging directions might require more allocentric representations, which could explain increased TMA in the temporal pole for the response-locked.

The retrosplenial cortex showed, as expected, higher TMA for the direction condition in the response-locked. These results are compatible with findings from Lin et al. (2015), who also observed increases in theta band during direction processing. This also might reflect the retrosplenial cortex translation between egocentric and allocentric reference frames (Vann et al., 2009). Indeed, our task involved a more egocentric representation for the question-locked, while the response-locked was probably easier to solve with an allocentric representation or a combination of the two, especially for the direction condition.

It should be noted that we did not observe significant differences in the temporal, frontal, parahippocampal, and parietal regions during the response-locked between the distance and direction condition. A possible explanation would be that these regions contribute in a very similar way to the processing of distance and direction in the theta band. However, we did not analyze other frequencies, which could potentially reveal different processing of distance and direction for those regions.

## Conclusion

The aim of our study was to understand the role played by theta waves in the cognitive map, especially how the cognitive map is recalled and how the manipulation of distances and directions within the cognitive map occurs. Our task taps into several cognitive abilities such as working memory, mental imagery, recall of long-term memories, and spatial orientation. This study seems to reveal the role of theta waves as a marker of direction processing in specific brain regions when manipulating spatial information, such as the retrosplenial cortex and the temporal poles. Increases of TMA in frontal, parahippocampal, temporal, and temporal pole regions seem to be markers of working memory and recall of the cognitive map. Therefore, our CMRT could be useful to test for direction difficulties in patients, as it is in line with the previous work of Descloux and Maurer (2018). Our study also shows that there are two distinct parts when testing the cognitive map: recall and manipulation of spatial information. This is often seen as two similar processes in literature, but our study demonstrates that these processes might be different, with theta waves from different brain regions contributing to either recall or manipulation and should be considered in further studies.

Our study has limitations. First, we were focused on theta frequencies, mainly due to the involvement of theta waves in spatial navigation. Further investigations could also include other frequencies to determine their role in the spatial navigation of familiar environments. However, at a technical level, the implementation of individual MRI would increase the accuracy of source localization, especially in small brain regions, such as the retrosplenial cortex. Finally, it would also be important to assess the role of gender differences in the electrophysiological activation of familiar environments, as shown by Castillo et al. (2021).

## Data availability statement

The raw data supporting the conclusions of this article will be made available by the authors, without undue reservation.

## Ethics statement

The studies involving human participants were reviewed and approved by Commission Éthique de la Faculté de Psychologie et des Sciences de l'Éducation. The patients/participants provided their written informed consent to participate in this study.

## Author contributions

MTA and KG performed the EEG recordings. NB and IF were involved in planning and supervision of the study. MTA, KG, and NB processed the experimental data, performed the analysis, drafted the manuscript, and designed the figures. MS performed the source analysis calculations. IF and RM aided in interpreting the results and worked on the manuscript. All authors edited the draft, discussed the results, and commented on the manuscript. All authors contributed to the article and approved the submitted version.
