# Supplementary figures and images for: Electrophysiological correlates of distance and direction processing during cognitive map retrieval: A source analysis
Source: Front Hum Neurosci. 2023 Feb 22;17:1062064. doi: 10.3389/fnhum.2023.1062064 (PMC9992539; doi:10.3389/fnhum.2023.1062064)

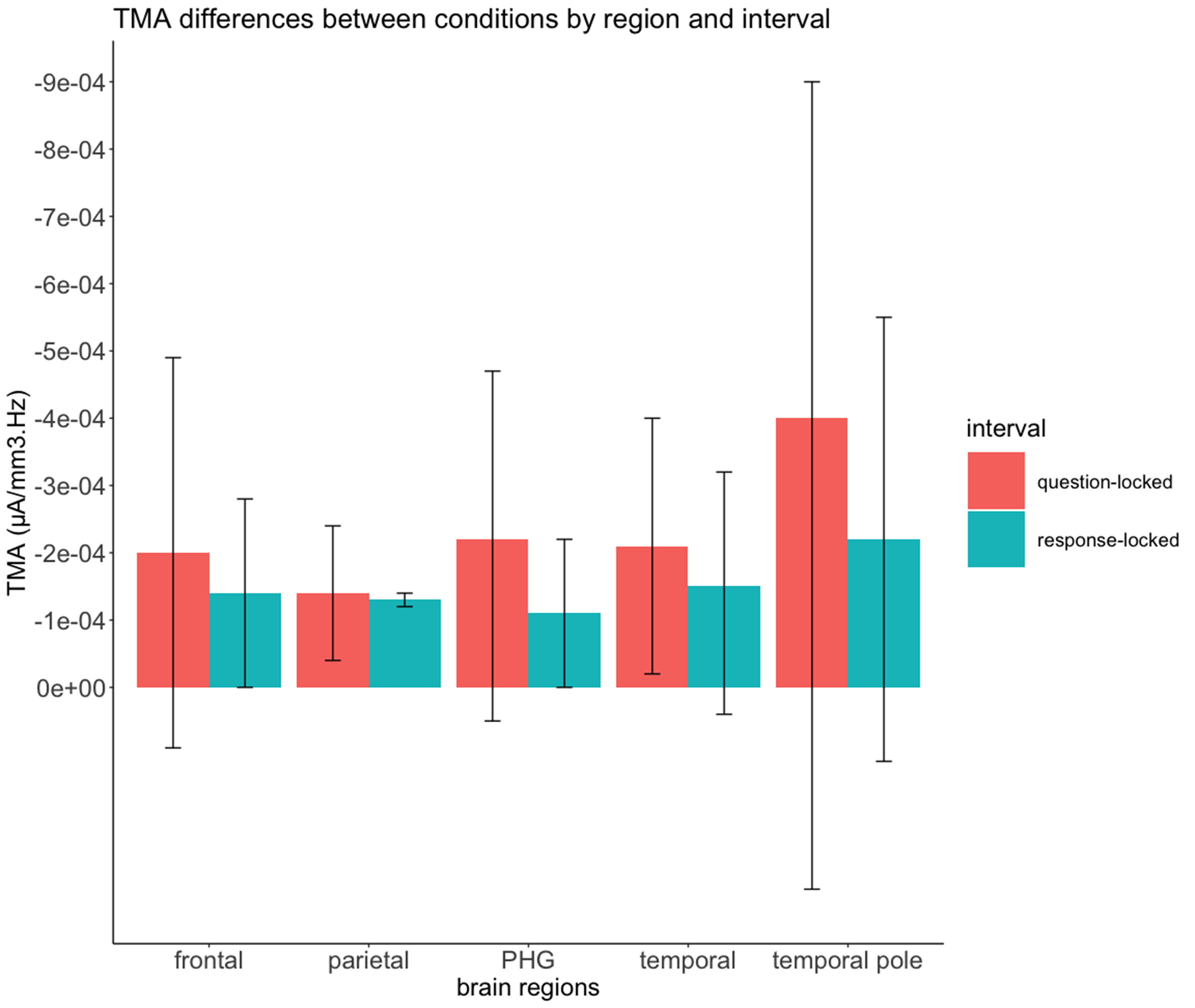

Supplement: Supplementary file 1 [file Image_1.png]
