# Supplementary material for: Electrophysiological correlates of distance and direction processing during cognitive map retrieval: A source analysis
Source: Front Hum Neurosci. 2023 Feb 22;17:1062064. doi: 10.3389/fnhum.2023.1062064 (PMC9992539; doi:10.3389/fnhum.2023.1062064)
Supplement: Supplementary file 2 [file Table_1.pdf]

Robust Paired Samples T-Test

		t	df	p
parietal left question distance	parietal left question direction	-5.54	17.0	< .001
parietal right question distance	parietal right question direction	-8.97	17.0	< .001
temporal left question distance	temporal left question direction	-6.83	17.0	< .001
temporal right question distance	temporal right question direction	-5.16	17.0	< .001
temporal pole left question distance	temporal pole left question direction	-7.82	17.0	< .001
temporal pole right question distance	temporal pole right question direction	-6.47	17.0	< .001
parahippocampus left question distance	parahippocampus left question direction	-6.03	17.0	< .001
parahippocampus right question distance	parahippocampus right question direction	-5.51	17.0	< .001
frontal left question distance	frontal left question direction	-5.84	17.0	< .001
frontal right question distance	frontal right question direction	-5.02	17.0	< .001
parietal left response distance	parietal left response direction	-5.43	17.0	< .001
parietal right response distance	parietal right response direction	-6.88	17.0	< .001
temporal left response distance	temporal left response direction	-4.12	17.0	< .001
temporal right response distance	temporal right response direction	-4.76	17.0	< .001
temporal pole left response distance	temporal pole left response direction	-3.02	17.0	0.008
temporal pole right response distance	temporal pole right response direction	-2.57	17.0	0.020
parahippocampus left response distance	parahippocampus left response direction	-4.07	17.0	< .001
parahippocampus right response distance	parahippocampus right response direction	-4.03	17.0	< .001
frontal left response distance	frontal left response direction	-7.28	17.0	< .001
frontal right response distance	frontal right response direction	-4.04	17.0	< .001
