# Supplementary material for: Electrophysiological correlates of distance and direction processing during cognitive map retrieval: A source analysis
Source: Front Hum Neurosci. 2023 Feb 22;17:1062064. doi: 10.3389/fnhum.2023.1062064 (PMC9992539; doi:10.3389/fnhum.2023.1062064)
Supplement: Supplementary file 3 [file Table_2.pdf]

Paired Samples T-Test

			Statistic	df	p
parietal left question distance	parietal left question direction	Student's t	-5.47	29.0	< .001
		Wilcoxon W	0.00		< .001
parietal right question distance	parietal right question direction	Student's t	-10.76	29.0	< .001
		Wilcoxon W	0.00		< .001
temporal left question distance	temporal left question direction	Student's t	-5.12	29.0	< .001
		Wilcoxon W	0.00		< .001
temporal right question distance	temporal right question direction	Student's t	-6.93	29.0	< .001
		Wilcoxon W	0.00		< .001
temporal pole left question distance	temporal pole left question direction	Student's t	-3.26	29.0	0.003
		Wilcoxon W	22.00		< .001
temporal pole right question distance	temporal pole right question direction	Student's t	-3.13	29.0	0.004
		Wilcoxon W	7.00		< .001
parahippocampus left question distance	parahippocampus left question direction	Student's t	-5.12	29.0	< .001
		Wilcoxon W	0.00		< .001
parahippocampus right question distance	parahippocampus right question direction	Student's t	-4.10	29.0	< .001
		Wilcoxon W	0.00		< .001
frontal left question distance	frontal left question direction	Student's t	-3.68	29.0	< .001
		Wilcoxon W	3.00		< .001
frontal right question distance	frontal right question direction	Student's t	-3.85	29.0	< .001
		Wilcoxon W	3.00		< .001
parietal left response distance	parietal left response direction	Student's t	-5.74	29.0	< .001
		Wilcoxon W	0.00		< .001
parietal right response distance	parietal right response direction	Student's t	-10.22	29.0	< .001
		Wilcoxon W	0.00		< .001
temporal left response distance	temporal left response direction	Student's t	-3.48	29.0	0.002
		Wilcoxon W	27.00		< .001
temporal right response distance	temporal right response direction	Student's t	-5.85	29.0	< .001
		Wilcoxon W	11.00		< .001
temporal pole left response distance	temporal pole left response direction	Student's t	-3.61	29.0	0.001
		Wilcoxon W	19.00		< .001
temporal pole right response distance	temporal pole right response direction	Student's t	-3.63	29.0	0.001
		Wilcoxon W	26.00		< .001
parahippocampus left response distance	parahippocampus left response direction	Student's t	-5.45	29.0	< .001

Paired Samples T-Test

			Statistic	df	p
		Wilcoxon W	13.00		<.001
parahippocampus right response distance	parahippocampus right response direction	Student's t	-5.54	29.0	<.001
		Wilcoxon W	10.00		<.001
frontal left response distance	frontal left response direction	Student's t	-5.56	29.0	<.001
		Wilcoxon W	0.00		<.001
frontal right response distance	frontal right response direction	Student's t	-4.82	29.0	<.001
		Wilcoxon W	25.00		<.001
